# Editorial: Advances in the diagnosis and genomic research of surveillance-response activities in emerging, re-emerging, and unidentified infectious diseases

**DOI:** 10.3389/fpubh.2023.1182918

**Published:** 2023-05-23

**Authors:** Chenxi Wang, Xinyu Feng, Jun Feng, Hui Chen, Tianmu Chen, Kun Yin

**Affiliations:** ^1^School of Global Health, Chinese Center for Tropical Diseases Research, Shanghai Jiao Tong University School of Medicine, Shanghai, China; ^2^One Health Center, Shanghai Jiao Tong University-The University of Edinburgh, Shanghai, China; ^3^National Institute of Parasitic Diseases, Chinese Center for Disease Control and Prevention (Chinese Center for Tropical Diseases Research), Shanghai, China; ^4^NHC Key Laboratory of Parasite and Vector Biology, Shanghai, China; ^5^World Health Organization Collaborating Centre for Tropical Diseases, Shanghai, China; ^6^National Center for International Research on Tropical Diseases, Shanghai, China; ^7^Department of Biology, College of Life Sciences, Inner Mongolia University, Hohhot, China; ^8^Shanghai Municipal Center for Diseases Control and Prevention, Shanghai, China; ^9^Brigham and Women's Hospital, Harvard Medical School, Cambridge, MA, United States; ^10^State Key Laboratory of Molecular Vaccinology and Molecular Diagnostics, School of Public Health, Xiamen University, Xiamen, Fujian, China

**Keywords:** surveillance, infectious diseases, diagnosis, genomic research, public health

Six pandemics of public health emergencies were announced as a global concern, causing numerous deaths and economic losses in this century (1). Strikingly, more than 3.5 million people died from COVID-19 in 2021, exceeding the combined number of deaths resulting from HIV, malaria, and tuberculosis in 2020 (2). The delayed diagnosis and inadequate surveillance of infectious diseases may indirectly lead to ineffective responses, making it harder to control the development of pandemics. Despite advances in health science, infectious diseases, including emerging, re-emerging, and unidentified infectious diseases, remain a major threat to human beings, particularly in low-resource settings.

Recent studies has been made in understanding infectious diseases transmission and dynamics (3). However, there are still gaps between diagnostic capacity and dynamic surveillance of infectious diseases. Nowadays, researchers have made significant efforts to optimize surveillance performance to emerging, reemerging, and unidentified infectious diseases, including diagnosis and genomic research (4, 5). However, increasing efforts are highly required to develop a more effective surveillance technique to continuously monitor, early warning and predict infectious diseases.

Therefore, we focused on the advances in the diagnosis and genomic research of surveillance-response activities in emerging, re-emerging, and unidentified infectious diseases in this Research Topic, which will contribute to infectious disease prediction and the effective decision making. Eleven papers were published worldwide, including opinion articles, original research, and case reports.

## Apply metagenomics and molecular methods to combat infectious diseases

Currently, metagenomic surveillance provides an opportunity to improve the detection and prevention of pathogens by conducting epidemiological investigations (Zhang T. et al.), limiting clonal transmission of bacteria (Chen et al.), and ensuring the effectiveness of interventions (Zeng et al.). Padilla-Blanco et al. described a new variant of SARS-CoV-19 that emerged in Sicily in late 2020. It showed that continuous monitoring of SARS-CoV-2 varirants is helpful in understanding virus evolution and provides timely information on sudden pathogen emergence, especially in times and places where epidemic virus pedigree is seldom explored. More importantly, monitoring viral variants by molecular biology technology can play an significant role in developing appropriate rapid detection tools and predicting potential vaccine escapes in emerging variants. For example, Feng et al. reported a case of *Parvimonas micra* pneumonia in a patient without obvious underlying disease and distal site of infection, expanding the etiology of pneumonia. It demonstrated that metagenomic sequencing allows rapid screening for rare pathogens, especially when regular strategy is ineffective. In addition, Tian et al. continuously monitored the changes in pathogenic bacteria by sequencing and drug sensitivity tests in a case of intracranial infection caused by multidrug-resistant *Acinetobacter baumannii*. As a complication of a series of invasive operations, the infection may seriously affect the prognosis of patients, in which it is necessary to monitor the pathogens in the patients' blood.

## Data-based approaches to combat infectious disease

Rapid advances in Big Data and machine learning provide opportunities to collect and deeply utilize epidemiological data for combating infectious diseases (6–9). Hand-foot-and-mouth disease (HFMD) is a common infectious disease in children caused by a variety of enteroviruses. Yang et al. explored the interaction between multiple viruses and the regular patterns of HFMD in different regions using the susceptibility-exposure-infection-asymptomatic-rehabilitation (SEIAR) model. HFMD is a common infectious disease in children caused by a variety of enteroviruses. The results showed that the incidence of HFMD increased with temperature risk, and the interaction of different pathogens strongly depended on geographic locations. Compared with the transmission of only one major subtype, there was a dilution effect when multiple subtypes of pathogens were transmitted simultaneously. Andagalu et al. conducted a cohort study to evaluate the efficacy of two antimalarial drugs, artemether-lumefantrine (AL) and artesunate-mefloquine (ASAQ), while developing predictive models of treatment outcomes based on immune profile data. Using machine learning to assess humoral immunity to malaria to predict the effectiveness of treatment, the researchers found that ASAQ was more effective. The investigators applied computational methods for the first time to show that serologic immune profiles differentially affect treatment outcomes based on artemisinin-based combination therapies (ACTs), providing an integrated approach to data integration, machine learning, and modeling.

## Promote the diagnosis and response to infectious diseases

Some rapid testing techniques, which have the advantage of rapid, low-cost, and user-friendly, are suitable for epidemiologic studies and show the potential as high-throughput screening tools. A recent study evaluated the rapid detection of anti-Trypanosoma cruzi antibodies, providing recommendations balanced between sensitivity and operability for large-scale investigations (Iturra et al.). In addition, based on previous research, two opinion articles provided some optimization suggestions for antigen detection of SARS-CoV-2 (Zhang J. et al.), and discussed the methods to treat or alleviate the symptoms of infectious diseases (Aydemir and Ulusu).

To effectively address the challenges from infectious disease outbreaks and public health emergencies, we need to strengthen surveillance on unexplained infectious diseases, improve the sensitivity and accuracy of diagnostic assessment, and enhance the capacity for *in situ* analysis and treatment. We expect that further studies on infectious diseases will lead to more cost-benefit programs that improve wellbeing and sustainability in diverse socio-ecological settings and ultimately throughout the world.

Thank all authors who contributed to this research theme, and we invite readers to explore the excellent articles in this compilation.

## Author contributions

KY contributed to the original idea and conceived the paper. CW wrote the initial draft of the paper. The final version was reviewed by XF, JF, TC, and HC. All authors contributed to the article and approved the submitted version.
